# A Biomimetic Collagen Derived Peptide Exhibits Anti-Angiogenic Activity in Triple Negative Breast Cancer

**DOI:** 10.1371/journal.pone.0111901

**Published:** 2014-11-10

**Authors:** Elena V. Rosca, Marie-France Penet, Noriko Mori, Jacob E. Koskimaki, Esak Lee, Niranjan B. Pandey, Zaver M. Bhujwalla, Aleksander S. Popel

**Affiliations:** 1 Department of Biomedical Engineering, The Johns Hopkins University School of Medicine, Baltimore, MD, 21205, United States of America; 2 Johns Hopkins University In-vivo Cellular and Molecular Imaging Center Program, Division of Cancer Imaging Research, The Russell H. Morgan Department of Radiology and Radiological Science, The Johns Hopkins University School of Medicine, Baltimore, MD, 21205, United States of America; 3 Department of Oncology and the Sidney Kimmel Comprehensive Cancer Center, The Johns Hopkins University School of Medicine, Baltimore, MD, 21205, United States of America; University of South Alabama, United States of America

## Abstract

We investigated the application of a mimetic 20 amino acid peptide derived from type IV collagen for treatment of breast cancer. We showed that the peptide induced a decrease of proliferation, adhesion, and migration of endothelial and tumor cells *in vitro*. We also observed an inhibition of triple negative MDA-MB-231 xenograft growth by 75% relative to control when administered intraperitoneally for 27 days at 10 mg/kg. We monitored *in vivo* the changes in vascular properties throughout the treatment using MRI and found that the vascular volume and permeability surface area product decreased significantly. The treatment also resulted in an increase of caspase-3 activity and in a reduction of microvascular density. The multiple mode of action of this peptide, *i.e*., anti-angiogenic, and anti-tumorigenic, makes it a viable candidate as a therapeutic agent as a monotherapy or in combination with other compounds.

## Introduction

Angiogenesis is a critical factor in tumor development, and anti-cancer therapies targeting angiogenesis are being extensively investigated [1]–[3]. These investigations have led to the clinical use of anti-angiogenic agents, such as the anti-vascular endothelial growth factor (VEGF) antibody Bevacizumab for breast and other cancers [4]–[6], and small molecule tyrosine kinase inhibitors such as Sunitinib [7]. The absence of overall survival increase has, however, resulted in the retraction of Bevacizumab as an anti-angiogenic therapy for breast cancer [8], leaving few other alternatives for anti-angiogenic treatment of breast cancer. Factors contributing to the failure of Bevacizumab are the limited understanding of its mode of action, and the paucity of biomarkers and techniques to continuously monitor treatment efficacy [9].

Peptides present an attractive alternative as anti-angiogenic agents, and several candidates which are currently in clinical trials are showing promising results [10], [11]. Peptide-based therapies offer multiple advantages. Their small size leads to good tumor penetration, they have high binding specificity, are flexible to modifications with different tags, and they can be conjugated with other drugs. Due to short biostability, modifications such as the introduction of non-natural amino acids or attachment to different backbones are being developed. By characterizing the structure activity relations (SAR), non-critical amino acids are identified and thus can be substituted by natural or non-natural amino acids to create more stable peptides while maintaining or even enhancing the overall potency [12]. We have identified many short peptides from endogenous human proteins with anti-angiogenic activity. SP2024, the peptide that we test here, was derived from a peptide originally identified in the non-collagenous domain of the α5 fibril of collagen IV after extensive structure activity relationship studies [13]. We have shown that earlier analogs of SP2024 have anti-angiogenic and anti-lymphangiogenic activities *in vitro* and *in vivo* [10], [14], [15]. We also showed that earlier SP2024 analogs bind to the extracellular domain of various integrins [10], [16]. Integrins serve as co-receptors for many different receptor tyrosine kinases that regulate angiogenesis such as VEGFR, FGFR, HGFR, IGFR [17], [18].

Anti-angiogenic treatments do not rapidly induce tumor shrinkage, and the ability to noninvasively detect changes in angiogenesis is critically important in the successful application of these treatments. Most markers used to investigate the effectiveness of anti-angiogenic therapies characterize terminal points of the treatment, such as microvascular density by immunohistochemistry (IHC) with anti-CD34 antibodies in tumor sections, thus no real time information of the efficacy response is available [19]. In the clinic, magnetic resonance imaging (MRI) is used for cancer diagnosis, prognosis, and to monitor treatment efficacy [20]. Recent studies have examined the use of dynamic contrast enhanced MRI to monitor the effects of anti-angiogenic therapy [21], [22]. In the present study, we used MRI to monitor noninvasively the changes in the tumor vasculature, thus gathering real time information on the efficacy of the peptide throughout the duration of the treatment. We used a high molecular weight contrast agent albumin-gadolinium-diethylenetriamine-pentaacetic acid (GdDTPA) [23] that can be applied to quantify tumor vascular volume and vascular permeability surface area product (PSP) [21]. To our knowledge, this is the first time that this method has been employed to monitor the effects of a peptide-based anti-angiogenic treatment. The method provides insights into the mode of action of this treatment, and can identify vascular parameters measurable with MRI that are most likely to detect therapeutic response in patients. The *in vivo* imaging results were correlated with *ex vivo* IHC staining of CD34 in tumor sections, indicative of the microvascular density. We also investigated the effect of our peptide on the proliferation, migration and adhesion of vascular cells, lymphatic endothelial cells (LEC), and tumor cells. Based on the changes observed following treatment of LEC *in vitro*, we investigated the changes in lymphatic vascular density in tumor sections, and found a reduction in lymphatic vascular density; however, the reduction did not reach the level of statistical significance. Our results identify SP2024 peptide as a promising new candidate for breast cancer therapy.

## Methods and Materials

### Cell culture

Human umbilical vein endothelial cells (HUVEC), and Human Lymphatic Endothelial Cells (LEC) were purchased from Lonza (Allendale, NJ). HUVEC were maintained using Endothelial Basal Media (EBM-2) supplemented with the Bullet Kit (Lonza, Allendale, NJ), the LEC were grown in Microvascular Endothelial Cell Growth Medium-2 (EGM-2MV) (Lonza, Allendale, NJ). Triple negative human breast cancer cells, MDA-MB-231, were provided by Dr. Zaver Bhujwalla. The breast cancer cells were grown in RPMI-1640 medium (Gibco, Carlsbad, CA) supplemented with 10 tm% fetal bovine serum (FBS) and antibiotics (1% penicillin and streptomycin). Cells were maintained under standard conditions of 37°C and 5% CO_2_, the passage numbers of the endothelial cells were between 2 and 7.

### Peptide synthesis

The peptide (denoted SP2024), SEQ: LRRFSTMPFMFININNVINF-NH_2_, and the scrambled peptide (denoted SP2048), SEQ: LRRFSTAPFAFIPEAKVINF-NH_2_ were synthesized using solid-phase synthesis and were supplied as a Trifluoroacetic (TFA) salt with an amidated C-terminus by New England Peptide (Gardner, MA). The purity of the peptides was >95% and the suppliers provided product characterization (MALDI-TOF and HPLC traces) for molecular weight and purity accuracy. The peptides stock was solubilized in 5% DMSO and water due to their hydrophobic profile. The pH of the solutions was measured before injection, and found to be around pH 7. For all *in vitro* experiments the DMSO % was maintained at non-toxic threshold (<0.2%) as determined by toxicity assays of DMSO on cells. The *in vivo* control contained the same DMSO % (5%), as the peptide solution.

### *In vitro* assays

#### Proliferation assay

Colorimetric-based proliferation assay using WST-1 proliferation reagent (Roche, Basel, Switzerland) was applied to HUVEC, LEC and MDA-MB-231 cells. 2000 cells/well were plated in 96-well plates and allowed to adhere overnight. The following day, the media were exchanged with fully supplemented media containing the SP2024 peptide or equivalent DMSO vehicle for the controls. Three days later, the media was replaced with serum-free EBM-2 media containing WST-1 reagent, and incubated for 4 hours at 37°C. The plates were read on a Victor V fluorescence plate reader (Perkin Elmer, MA) by measuring the absorbance at 450 nm.

#### Migration assays

The effect of the SP2024 peptide on cells migration was investigated using two different assays; a real time migration assay system based on electrical impedance (RT-CIM, ACEA Biosciences, CA) and a wound healing type assay (Oris Pro Migration assay, Platypus Technologies, CMA 1.101). The RT-CIM assay uses a CIM 16-well plate (Roche, Basel, Switzerland) composed of a top and bottom chamber separated by a microporous polycarbonate membrane (8 µm). The membrane was coated with fibronectin (20 µg/ml) and 45,000 cells/well (HUVEC or LEC) in serum free media, with or without peptide was added to the top compartment. Media with chemoattractant (*i.e*., fully supplemented EBM-2 or EBM-2MV) was added to the bottom compartment of the chamber, and the plate was incubated at 37°C for 20 hours. The sensors integrated on the bottom side of the membrane monitored and continuously recorded changes in impedance as the cells moved through the membrane. Breast cancer cells MDA-MB-231 are not suitable for RT-CIM type experiments due to their thin elongated phenotype that results in low signal, thus the inhibition of migration was investigated using a wound healing type assay. This assay was performed using the Oris Pro Migration assay (Platypus Technologies, CMA 1.101). Briefly, 25,000 cells/well in fully supplemented media were added to the 96-well plate containing stoppers that were used to block the migration of the cells to the center region of the wells. Cells were allowed to adhere for 4 hours, after which the stoppers were removed. After 18 hours cells were stained with Calcein AM (0.5 µg/ml) (Invitrogen, CA) and the cells that migrated to the center of the well were quantified by fluorescence intensity measured with a Victor V plate reader.

#### Adhesion assay

Similarly to the migration assays, the effect of the SP2024 peptide on cellular adhesion was assessed using the RT-CIM technology. In this case, 25,000 cells/well were plated in 16-well E-plates (Roche, Basel, Switzerland) in the presence or absence of the peptide. The adhesion was monitored over/for 3 hours) by measuring changes in the electrical impedance, which is a direct measure of the cells adhering on/to the electrodes. All *in vitro* studies used as control full supplemented media with equivalent DMSO concentration as in the treatment samples but maintained to levels less than 0.2%.

#### Integrin binding assay

One µg of 3 different integrin heterodimers (R&D Systems) was combined with 1 µg of SP2024 biotinylated at the N-terminus with LCBiotin (Pierce Biotechnology) in 500 µL Dulbecco’s PBS (DPBS) without Ca^2+^ and Mg^2+^ in the presence or absence of a 7X excess of unbiotinylated SP2024. The mixture was incubated at 4°C with end-over-end turning for 30 min. Ten µL of well mixed streptavidin sepharose (Cell Signaling Technology) was added and the mixture was incubated for another 30 min at 4°C. The reaction was spun down at 2000 rpm at room temperature, washed with 1 mL DPBS two times, 20 µL of LDS gel loading dye was added and electrophoresed after boiling. The gel was transferred to nitrocellulose membranes and probed with the appropriate anti-integrin antibody and secondary antibodies and detected using the GE chemiluminescence detection kit.

#### Western blot analysis

HUVECs grown in complete endothelial cell media were plated in tissue culture-treated 6-well plates at a density of 360,000 cells/well then serum-starved for 24 hours. The peptide SP2024 was added at 10 or 50 µM for 90 min. VEGF (Cell Signaling Technology, Inc., Danvers, MA) was then added at 20 ng/mL for 10 min. The reaction was stopped by adding cold PBS and lysis buffer (150 mM NaCl, 1 mM EDTA, 100 µL/ml protease inhibitors (Sigma, St. Louis, MO), 10 µL/ml phosphatase inhibitors (Sigma, St. Louis, MO) and 1% Triton) for 2 hours, and the cells were recovered by scraping. Cell lysates were spun at 14,000 g for 15 min to remove cell membranes and debris, separated by SDS-PAGE, and transferred to nitrocellulose blots (Invitrogen, Carlsbad, CA). Membranes were blocked for 1 hour with 5% milk and 1% BSA in TBST and probed with antibodies of interest in milk including anti-pVEGFR2, anti-phosphorylated PhospholipaseCγ (anti-pPLCγ), anti-PLCγ, and anti-GAPDH (Cell Signaling Technology, Inc). The next day, secondary antibodies were added at 1∶2000 dilutions, and protein bands were visualized with chemiluminescence detection reagent (GE Healthcare, United Kingdom). Blots were then stripped and probed for additional antibodies. Experiments were repeated at least once. Each blot shown is representative of one complete experiment.

### *In vivo* experiments

#### Ethics Statement

Animals were housed and treated according to the approved animal protocol of the Institutional Care and Use Committee at Johns Hopkins Medical Institutions (JHMI).

#### Tumor xenografts

Orthotopic breast tumors were initiated in severe combined immunodeficient (SCID) mice using MDA-MB-231 human triple negative breast cancer cells. 2×10^6^ cells in 100 µL were injected in the right upper thoracic mammary fat pad. Tumors reached volumes of 75 to 100 mm^3^ in approximately 14–21 days. Mice were randomized and arranged in groups (6–7 mice per group) with similar tumor volumes (no statistical difference among averages) and treatment was commenced. The SP2024 peptide was administered daily intraperitoneally (i.p.) at a dose of 10 mg/kg. The dose was chosen based on our previous work using the parent compound and the same *in vivo* model tumors [24]. Tumors were measured every fourth day using calipers and the tumor volume was calculated using the formula V = *ab* ^2^/2, where *a* is the larger and *b* is the smaller diameter. The control group received PBS with 5% DMSO (solubilization vehicle of the treatment group).

#### Magnetic resonance imaging and data processing

All imaging studies were done on a 9.4T Bruker spectrometer (Billerica, MA) using a home-built solenoid coil placed around the orthotopic tumors. Mice were anesthetized with an i.p. injection of ketamine (25 mg/kg; Phoenix Scientific, Inc., St Joseph, MO) and acepromazine (2.5 mg/kg; Aveco, Phoenix Scientific, St Joseph, MO) diluted in saline. The tail vein was catheterized before placing the animal in the spectrometer. Vascular imaging was performed as previously described [25]. Briefly, multislice relaxation rate (1/T_1_) maps were obtained on 4 slices (1 mm thick) with an in-plane spatial resolution of 125 µm (128×128 matrix, 16 mm field of view, 8 averages) by a saturation recovery method combined with fast T_1_ SNAPSHOT-FLASH imaging (flip angle of 10°, echo time of 2 ms). First, M_0_ maps with a recovery delay of 10 s were acquired following which images were obtained for three relaxation delays (100 ms, 500 ms, and 1 s). These T_1_ recovery maps were obtained before i.v. administration of 0.2 ml of 60 mg/ml albumin-GdDTPA in saline (dose of 500 mg/kg) and repeated over a 21 minute period, starting 3 minutes after the injection. The contrast agent albumin-GdDTPA was synthesized based on the method of Ogan *et al*. [23]. At the end of the imaging studies, the T_1_ of blood was measured. The mice were scanned at days 0, 10, 20 and 27 post-treatment. Relaxation maps were reconstructed from datasets for the three different relaxation times and the M_0_ dataset on a pixel-by-pixel basis. Vascular volume and PSP maps were generated from the ratio of (1/T_1_) values in the images to that of blood. The slope of (1/T_1_) ratios *versus* time in each pixel was used to compute PSP, and the intercept of the line at zero time was used to compute vascular volume. Data were processed with an operator-independent computer program that enabled selection, mapping, and display of the regions with a routine written using Interactive Data Language (IDL, Research Systems, Boulder, CO).

#### Immunohistochemistry

Following the sacrifice of the animals, tumors and muscle tissues from the hind leg were excised, stored in IHC zinc fixative (BD Biosciences, San Jose, CA) for 10 to 14 days, and processed by an outside contractor (Covance, Greenfield, IN). Briefly, tissues were embedded in paraffin and 5 µm sections were collected from the central cross-sectional area of the tissues. Following deparaffinization, CD34, LYVE-1 and caspase-3 staining was performed overnight. The staining was quantified using FRiDA software (Johns Hopkins University, Baltimore, MD). Pixels representative of the staining were isolated from the image using masking and subsequently counted. The number of pixels per image was compared among the conditions and the Student’s t-test was performed to assess statistical significance.

#### Data analysis

Statistical significance (*p*<0.05) within each experiment was assessed using statistical assessments such as Student’s t-tests or other appropriate statistical tests.

## Results

### *In vitro* activity

**Figure 1:**
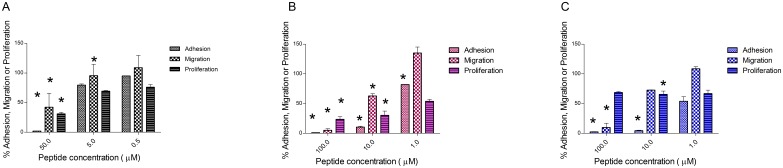
*In vitro* inhibition activity of the peptide on vascular endothelial cells, tumor cells and lymphatic endothelial cells. Inhibition of migration (checkered), adhesion (hashed) and proliferation (horizontally hashed) relative to the vehicle control, as a result of incubation of HUVEC (A), LEC (B), and tumor cells (C) with different concentrations of SP2024. Asterisks indicate statistical significance (p<0.05) in comparison to the treatment with the lowest concentration (0.5 or 1.0 µM).

The peptide SP2024 was developed from substitutions made on a parent peptide derived from the non-collagenous domain of collagen IV, identified *via* a bioinformatics study [26]. These modifications were introduced to increase the solubility and the stability of the peptide, and resulted in an increased potency over the original peptide. Figure 1 is a compilation of the various inhibitory activities of SP2024. We investigated the effect of the peptide on processes key to tumor progression, angiogenesis, and lymphangiogenesis: proliferation, adhesion and migration. We examined these effects in three different cell lines: HUVEC for anti-angiogenic activity, LEC for anti-lymphangiogenic activity, and MDA-MB-231 tumor cells for anti-tumorigenic activity. Figure 1A illustrates the percent inhibition relative to the vehicle control in migration, adhesion and proliferation of HUVEC by SP2024, showing its anti-angiogenic activity. As shown in Figure 1B, SP2024 also shows statistically significant inhibition of LEC migration, adhesion and proliferation, although a higher concentration of peptide is necessary to observe a level of inhibition comparable to that of inhibition of HUVEC. Figure 1C illustrates the anti-tumorigenic activity of the peptide with a strong inhibition of MDA-MB-231 cell migration and adhesion, but a minimal inhibition of cell proliferation.

**Figure 2:**
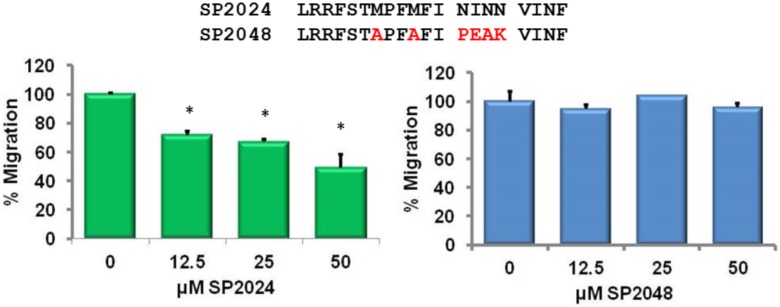
Specific migration inhibition on Microvascular Endothelial Cells (MECs). Inhibition of migration by SP2024 is dose dependent (panel A) while the scrambled peptide SP2048 (panel B) showed no effect on migration irrespective of the treatment dose. Asterisks indicate statistical significance (p<0.05) in comparison to the control.

The activity of the scrambled peptide was investigated in the migration of microvascular endothelial cells (MEC) and as shown in Figure 2 the scrambled peptide has no effect on migration, while at the same concentrations SP2024 significantly inhibits migration in a dose dependent manner.

**Figure 3:**
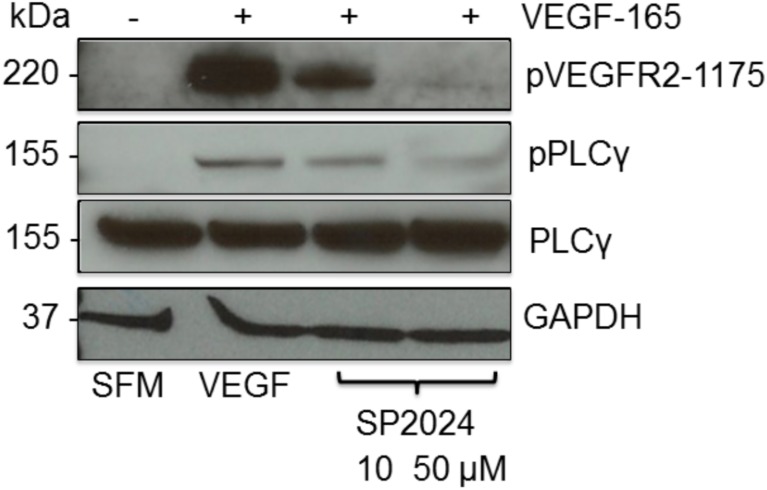
Inhibition of the phosphorylation of signaling proteins of the VEGFR2 pathway. Western blot analysis of the effect of the peptide treatment of HUVEC cells on the phosphorylation of VEGFR2 and PLCγ. The first column represents the base level of proteins expression in the presence of serum free media (SFM), the second column shows the levels of the proteins when cells are treated with VEGF (20 ng/ml) and the next two columns show the decrease in the levels of the proteins in cells exposed to VEGF (20 ng/ml) and 10 or 50 µM of SP2024. GAPDH was used as a loading control.

To further understand the mechanism by which SP2024 inhibits cell migration, proliferation and adhesion, we investigated the effect of SP2024 on different proteins involved in the VEGF-mediated signaling pathway which can affect all of these processes. We assessed the levels of phosphorylated VEGFR2, the main signaling pro-angiogenic receptor, along with PLCγ an upstream component of the signaling pathway, by western blot analysis of HUVEC lysates as illustrated in Figure 3. We found that treatment with SP2024 at 10 and 50 µM resulted in a significant reduction of the phosphorylated proteins indicating that the peptide acts specifically through the VEGFR2 pathway to inhibit the processes of proliferation and migration.

**Figure 4:**
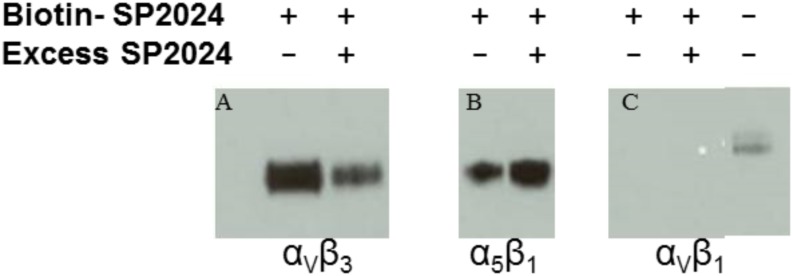
Binding of the peptides to the integrins. Specific binding of the biotinylated peptide to specific integrins involved in regulation of angiogenesis. Panel A shows the specific binding to the integrin α_V_β_3_, in contrast to non-specific binding to the α_5_β_1_ (panel B). Panel C indicates no binding to the α_V_β_1_ integrin.

Moreover, we investigated the binding of the peptide to the integrins known to be involved in angiogenesis, such as α_V_β_1,_ α_V_β_3_ and α_5_β_1_. The binding results illustrated in Figure 4 indicate that the peptide specifically binds to the α_V_β_3_ since the binding is reduced in presence of excess peptide; in contrast, binding to the α_5_β_1_ is non-specific since the binding is not affected by excess peptide. We also show that the peptide does not bind to the α_V_β_1_ as indicated in panel C of Figure 4 by the absence of a band.

### In vivo activity

**Figure 5:**
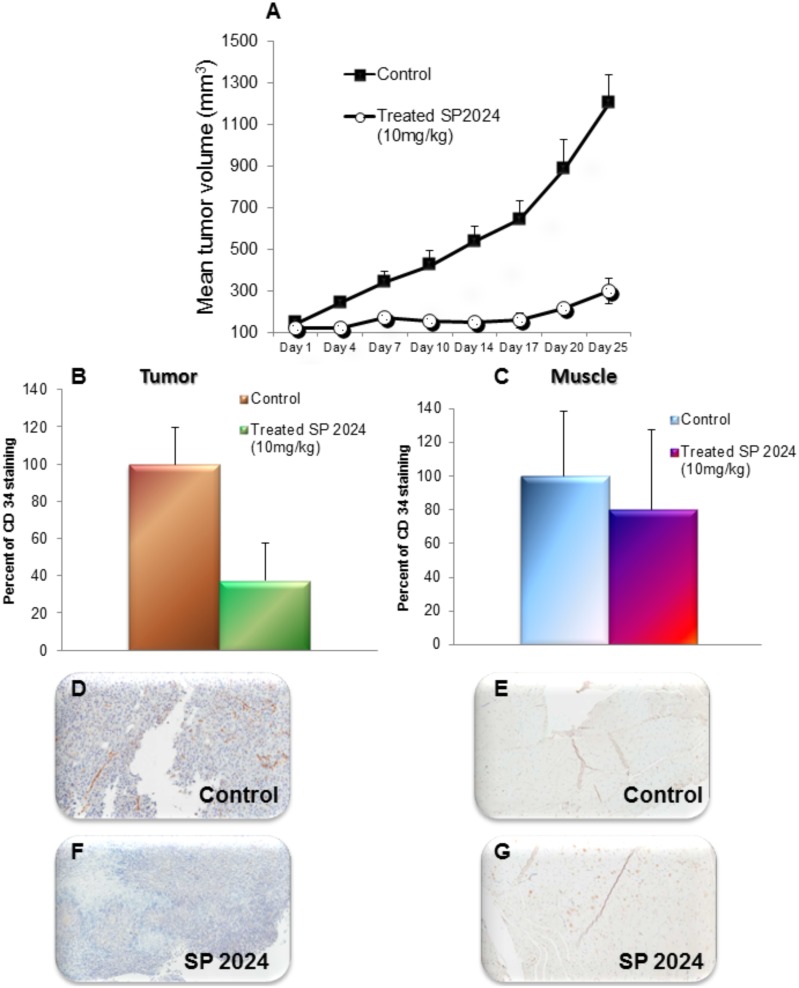
Tumor growth inhibition and effects on the density of microvascular density. Inhibition of growth of MDA-MB-231 orthotopic xenografts as a result of peptide treatment. When tumors reached a volume of approximately 100 mm^3^ treatment commenced with daily i.p. injections of 10 mg/kg SP2024. Tumor size was monitored by calipers every fourth day. Statistical significance (p<0.001) between the treated and control groups was observed starting (A) with Day 7 measurement and continued until the termination of the study (Day 27). (B and C) Quantification of microvascular density in sections stained by IHC of tumors and muscles using CD34 antibodies showing significant differences (p<0.001) in the tumors but no significant difference in muscle (p = 0.093), illustrating the specificity of the peptide to neovasculature. (D, E, F, and G) Representative images of tumors and muscles from the control and treated groups.

Figure 5A illustrates the inhibition of MDA-MB-231 tumor growth with a daily intraperitoneal (i.p.) injection of SP2024 (10 mg/kg) for 27 days. This treatment resulted in a tumor growth inhibition of 75% in comparison to the untreated group (*p*<0.001). The parent peptide was investigated at a range of concentrations and different methods of administration (i.p. vs subcutaneous) and based on these results we chose to investigate the i.p. route of administration (most effective in the previous study) and a dose of 10 mg/kg representative of a median response.

To further confirm the anti-angiogenic activity of the peptide, we performed IHC on excised tumors at day 27 post-treatment. Microvascular density, a good indicator of anti-angiogenic activity [19], was assessed by staining for CD34, a marker of active angiogenesis. This staining is a good indicator of the effect of the treatment on tumor angiogenesis. We observed a 60% reduction in microvascular density as a result of the treatment (*p*<0.05) (Figure 5B). Figures 5D and 5F show representative images of CD34 staining on a control and a treated tumor respectively. Quantification using absolute number of pixels stained with CD34 are presented in the Figure (S1).

Anti-angiogenic treatments will fulfill their potential of specificity only if they affect the neovasculature, leaving the remaining vasculature intact, thus avoiding potential side effects of damaging the established vasculature. To assess the specificity of our SP2024 peptide, we investigated the effect of the treatment on muscle excised from the hind limb. The results are illustrated in Figures 5C, E, and G. Figure 5C shows that there was no statistically significant difference in the CD34 staining of the muscle tissues between the control and the treated animals. Figures 5E and G show representative CD34 staining of muscle tissues from a control and a treated animal respectively, displaying no significant difference.

**Figure 6:**
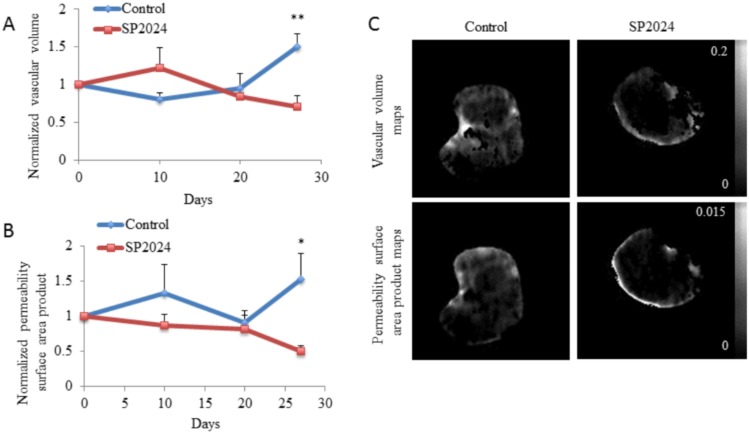
Vascular Volume and Permeability Surface Area Product. Vascular volume and vascular PSP measurement using albumin-GdDTPA contrast MRI. (A) Changes in vascular volume as a result of the daily i.p. injections with SP2024, with significant difference (p<0.01) on day 27. (B) Changes in the vascular PSP with significant difference (p<0.05) on day 27. (C) Representative vascular volume and PSP maps for the SP2024 treated groups in comparison to the control group.

It has been shown that anti-angiogenic treatments can improve tumor vascularization temporarily by pruning away some of the extensive branching of the new vasculature, thus leading to a normalization of the vasculature [27]. This coupled with a final reduction in vascular volume should lead to a change in vascular permeability. We explored the effect of the SP2024 peptide on MDA-MB-231 tumor vasculature using MRI. With the injection of the contrast agent albumin-GdDTPA vascular volume and permeability-surface area product (PSP) can be characterized *in vivo* noninvasively. We found, in the treated group, a significant reduction in the tumor vascular PSP (*p*<0.05) along with a significant reduction in the tumor vascular volume (*p*<0.01) at day 27 post-treatment (Figures 6A and B). Representative tumor vascular volume and PSP maps are displayed in Figure 6C.

**Figure 7:**
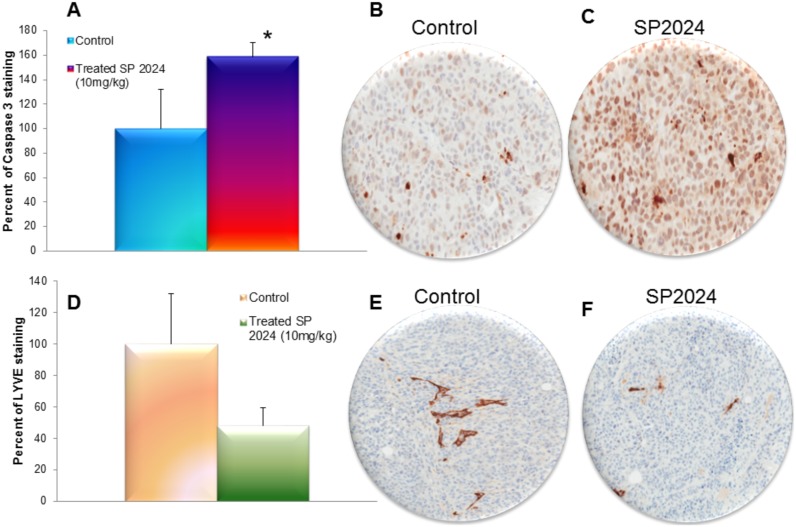
Caspase-3 and lymphatic vessels quantification. Assessment of the levels of caspase-3 in control and SP2024-treated tumor sections. (A) Significant increase (p<0.05) of caspase-3 in the treated tumors in comparison to the untreated tumors (B and C). Representative images of untreated tumor section (B) and treated tumor section (C). (D) Quantification of the lymphatic vasculature in the control group in comparison to the treated group. There was no statistical significance due to the scarcity of lymphatic vasculature but the difference between the two groups was visible on tumor sections. (E and F) Representative images of the control and the treated group respectively.

*In vitro* results showed that SP2024 inhibited the proliferation of both HUVEC and MDA-MB-231 tumor cells. We then assessed the level of apoptosis in the excised tumor sections by caspase-3 staining. We observed a significant increase in caspase-3 activity in the SP2024 treated group compared to the control group. Figure 7 shows the caspase-3 staining and quantification; the treatment resulted in a 60% increase of caspase-3 activity in the treated tumor sections compared to the control ones. This result was indicative of an increased apoptotic phenotype due to the treatment that could explain the significantly reduced tumor growth.

Since *in vitro* treatment with the SP2024 peptide resulted in statistically significant inhibition of proliferation, adhesion and migration of LEC, we measured the effect of the peptide on the lymphatic vessels in the MDA-MB-231 treated tumor by staining for the molecular marker LYVE-1 in the excised tumor sections [28]. Figure 7D shows the quantification of LYVE-1 in the stained sections, which indicate a decrease in the lymphatic vessel density. Figures 7E and F show representative images of the staining of LYVE-1 marker in control and treated tumor sections respectively. However, possibly since lymphatic vasculature is scarcer than blood vasculature and more variable, the reduction was not statistically significant (*p* = 0.093).

## Discussion

In the present study, we investigated the effect of an anti-angiogenic peptide on tumor progression. This peptide is an optimized version of a peptide derived from the non-collagenous domain of collagen IV [24]. The replacement of the two cysteine residues by isoleucine resulted in greater solubility, stability and activity over the parent peptide [13]. We studied the effect of the modified SP2024 peptide on tumor growth by treating MDA-MB-231 tumor bearing mice daily with i.p. injections, and we observed an inhibition of tumor growth. To understand the mechanism of action of the peptide, we investigated its effect on several processes that are critical for tumor progression, and found that the peptide significantly inhibited proliferation, migration and adhesion not only of vascular cells, but also of tumor cells. We then showed that this inhibition was mediated *via* the VEGFR2 pathway by demonstrating inhibition of phosphorylation of VEGFR2 in the presence of the peptide and that of a downstream molecule PLCγ in endothelial cells, while in cells which do not express VEGFR2 in vitro such as MDA-MB-231 [29], the effects shown in Figure 1C are likely the results of the integrin inhibition and are not VEGFR2 dependent. We also demonstrated specific binding of SP2024 to the integrin α_V_β_3_, in contrast to non-specific binding to the α_5_β_1_ and no binding to the α_V_β_1_ integrin. While these results demonstrate the effects of the peptide on the VEGFR signaling and integrin binding they do not reveal the full mechanistic detail of the signaling crosstalk between the integrins and VEGFR2. This crosstalk has been investigated in the last decade and is discussed in a number of publications [30]–[33]. Moreover, the *in vitro* activity that we observed on treated cells translated *in vivo* into a significant increase in caspase-3 activity in the treated tumors, and by a significant reduction in the microvascular density that can be explained by a decreased proliferation, migration and adhesion of endothelial cells. Finally, we observed changes in the vascular volume and the vascular PSP throughout the duration of the treatment using MRI. We showed that the treatment with this anti-angiogenic peptide resulted in a significant decrease of vascular volume and PSP after four weeks of treatment. The vascular permeability curve has a continuous decreasing trend throughout the duration of the treatment with significant decrease by day 27.

After investigating the activity of the peptide on endothelial cells, we have tested its effect on lymphatic endothelial cells. *In vitro* results have shown a reduced potency on LEC in comparison to the activity on vascular endothelial cells. We investigated if this activity correlates to an effect *in vivo* by assessing the density of lymphatic vessels in tumor sections and found a decrease in their density; however, this decrease did not reach the level of statistical significance.

Since this peptide is derived from collagen IV, we also investigated its effect on the expression of other extracellular proteins, such as collagen I, and found no significant effect (results not shown).

Our study showed that the SP2024 peptide is a strong candidate for cancer therapy owing to its multimodal activity. By inhibiting the proliferation, adhesion and migration of vascular and lymphatic endothelial cells, the peptide caused a reduction in microvascular and lymphatic vessel densities. Combined with its anti-tumorigenic effects, the treatment resulted in an inhibition of tumor growth *in vivo*. Moreover, the treatment affected, specifically, the neovasculature and not the established vasculature, as demonstrated by the absence of decrease in the vasculature of muscle tissue from treated mice, making the SP2024 peptide a promising candidate for anti-angiogenic therapy in breast cancer.

Anti-angiogenic therapies have not yet fulfilled the hopes that existed at their discovery, partly due to the inability to monitor their activity over the course of the treatment. Implementing a monitoring regimen as described in this study would allow physicians to adjust the dose or frequency of the treatment in the future, which might increase the success and efficacy of such therapies.

## Conclusion

In this study we investigated the application of a peptide derived from type IV collagen for the treatment of breast cancer. *In vitro* studies demonstrated that the peptide inhibits proliferation, adhesion, and migration of endothelial and tumor cells. *In vivo* studies showed a significant growth inhibition of triple negative MDA-MB-231 xenografts. Changes in vascular properties throughout the treatment were monitored using MRI which showed that the vascular volume and permeability surface area product decreased significantly. In *ex vivo* immunohistochemistry of the tumors we showed that the treatment also resulted in an increase of caspase-3 activity and in a reduction of microvascular density. The anti-angiogenic, anti-tumorigenic mode and possibly anti-lymphangiogenic action of this peptide, makes it a viable candidate as a therapeutic agent as a monotherapy or in combination with other therapeutic approaches.

## Supporting Information

Figure S1 **CD34, Caspase-3 and lymphatic vessel quantification.** Quantification of the absolute number of stained pixels. (A) CD34 staining in tumor, (B) CD 34 staining in muscle, (C) LYVE-1 staining in tumor and (D) Caspase 3 staining in tumor.
(TIF)
