# Supplementary figures and images for: A Biomimetic Collagen Derived Peptide Exhibits Anti-Angiogenic Activity in Triple Negative Breast Cancer
Source: PLoS One. 2014 Nov 10;9(11):e111901. doi: 10.1371/journal.pone.0111901 (PMC4226498; doi:10.1371/journal.pone.0111901)

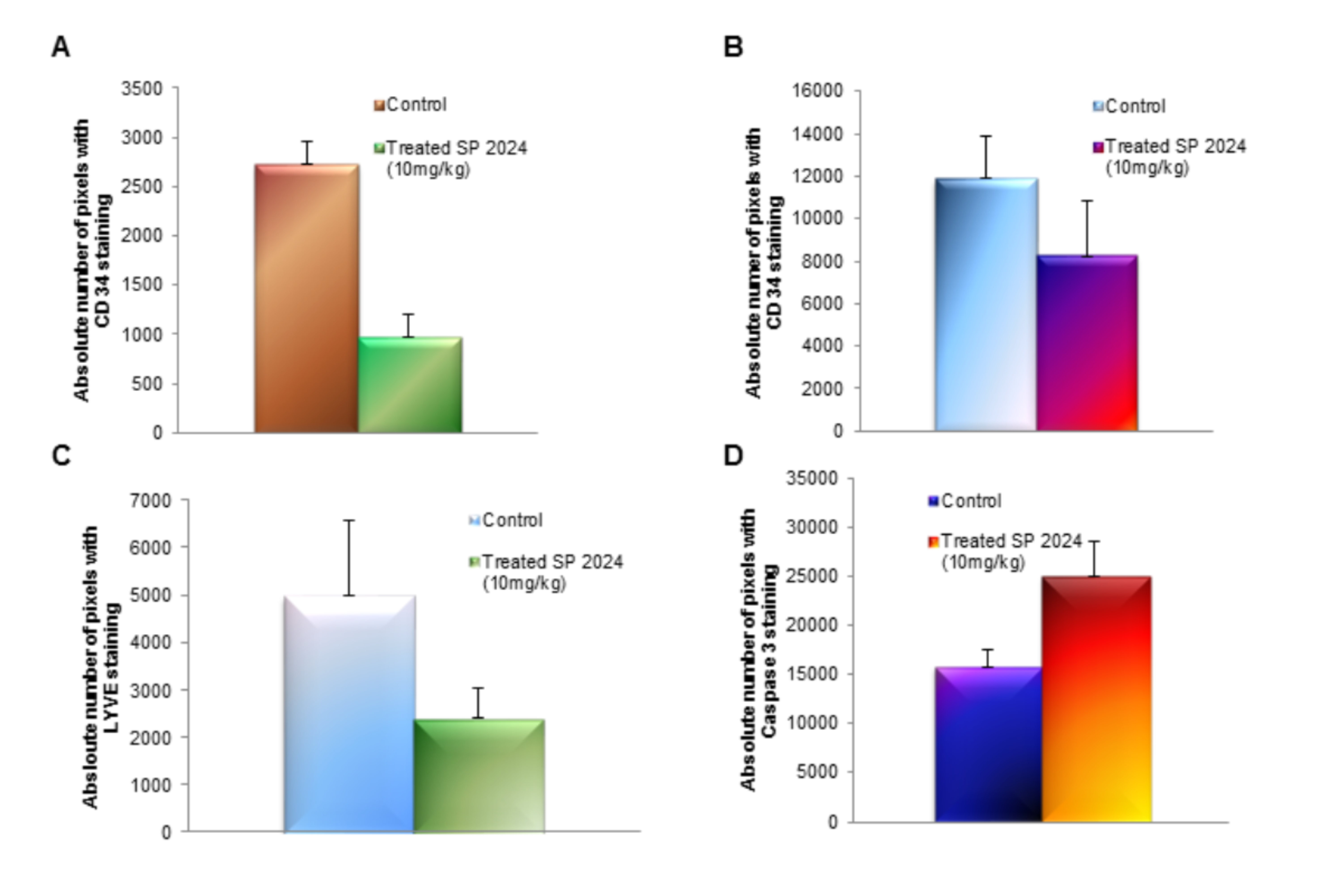

Supplement: Figure S1 — CD34, Caspase-3 and lymphatic vessel quantification. Quantification of the absolute number of stained pixels. (A) CD34 staining in tumor, (B) CD 34 staining in muscle, (C) LYVE-1 staining in tumor and (D) Caspase 3 staining in tumor. (TIF) [file pone.0111901.s001.tif]
